# Bridging Gaps: The Power of The Geriatrics 5M’s in Transforming Knowledge

**DOI:** 10.1093/geroni/igaf122.3157

**Published:** 2025-12-31

**Authors:** Ashmeet Bhatia, Keisha Velazquez-Diaz, Kamal Patel, James Meisel, Supriya Parvatini, Lionel Lim

**Affiliations:** VA Bedford Health Care System, Bedford, Massachusetts, United States; VA Bedford Health Care System, Bedford, Massachusetts, United States; VA Bedford Health Care System, Bedford, Massachusetts, United States; VA Bedford Health Care System, Bedford, Massachusetts, United States; VA Bedford Health Care System, Bedford, Massachusetts, United States; VA Bedford Health Care System, Bedford, Massachusetts, United States

## Abstract

Age-friendly care improves health outcomes for older adults. The Geriatrics 5M’s framework—Mind, Mobility, Medications, Multi-complexity, and Matters Most—serve as a guide to ensure that healthcare providers address the multifaceted needs of older patients. However, awareness and perceived application of these principles among hospital staff may be inconsistent. Literature reveals limited studies focusing on increased awareness and application after 5M’s workshop. VA Community Living Centers present opportunities for interprofessional education to identify gaps in knowledge and areas for improvement to foster a more age-friendly healthcare environment. This cross-sectional study aims to assess awareness and understanding of the Geriatrics 5M’s before and after an educational intervention across health professions, including nurses, physicians, trainees, and allied health professionals (n = 39). Staff were surveyed for knowledge before and after an educational intervention, which was delivered through workshops and learning materials. Results indicate a variable level of awareness of the 5Ms across health professions. Medical care providers showed higher familiarity compared to nurses and allied health professionals. There was improvement in knowledge of the 5M’s amongst physician trainees on geriatrics rotation at the VA and after a 5M’s workshop. There was increased knowledge across all groups using a Geriatrics 5M’s-focused educational curriculum, with notable increases in the “Medications” and “Matters Most” components. Our findings highlight the importance and impact of geriatric education using the Geriatrics 5M’s approach in improving interprofessional knowledge of age-friendly care. Further research is needed to explore the long-term impact of these educational efforts on health outcomes and patient care quality.

